# Addressing the migration of health professionals: the role of working conditions and educational placements

**DOI:** 10.1186/1471-2458-9-S1-S7

**Published:** 2009-11-18

**Authors:** Julia Witt

**Affiliations:** 1 Department of Economics, University of Manitoba, Winnipeg MB R3T 5V5, Canada

## Abstract

This article provides a brief overview of the global health-worker shortage, which could undermine the Millennium Development Goal to halt and begin to reverse the spread of HIV/AIDS. The current situation suggests that long-term solutions to shortages can only be found by addressing the problem from a global perspective; that is, to eliminate shortages through substantial investments in training and retaining health workers in developed and developing countries, and not through policies that do not work towards solving this underlying problem, such as ones that restrict migration.

## 

Goal number 6 of the Millennium Development Goals, "Combat HIV/AIDS and other diseases", aims to halt and begin to reverse the spread of HIV/AIDS by the year 2015. While some gains toward this objective have been made, the estimated number of people living with HIV globally continues to increase [1]. Without dramatic action, the MDG target is likely to become elusive.

Many advances have been made; for example, the number of people receiving antiretroviral drugs has increased five-fold in low- and middle-income countries between 2003 and 2006 [1]. However, major obstacles remain, including severe shortages of labour and infrastructure, which are the building blocks of comprehensive programs that will help achieve the MDG target. In a UNAIDS report [1], it is estimated that the number of full-time equivalents of health personnel needed to achieve the universal-access targets by 2010 are nearly half a million, though other sources place this estimate at twice that number [2]. Without reaching universal access by 2010, it is unlikely that goal number 6 will be achieved by 2015.

The shortage of health workers afflicts many countries around the world. Aiken et al. [3] show that, by 2011, the shortfall in the United States, the UK, Ireland, Canada, Australia and New Zealand that is predicted by each country's health-workforce-planning agency will, in combination, be well over 300,000 nurses. Even in the Philippines, where many more nurses are educated than are required for that country's needs, the effect of migration has been so strong that a domestic shortage is being created [4]. In other countries, including sub-Saharan Africa, migration is placing increasing pressure on fragile health systems that are already overburdened; nurse-to-patient ratios are as low as 10 per 100,000, compared to the UK, where the nurse-to-patient ratio is 847 per 100,000 [4]. The global shortage is likely to become exacerbated because the demand for nurses and other health professionals is growing faster than their supply.

Even though there is a lack of data to quantify the extent of migration [5,6], it is widely accepted that the 'brain drain' (the emigration of skilled workers) from Southern African and other developing countries is causing skill shortages in these countries, and that health professionals are among the largest professional occupational groups that are migrating. For the HIV/AIDS crisis in sub-Saharan Africa, this has translated into crippling effects on achieving health gains. Kober and Van Damme [7] paint a grim picture of the current situation in four African countries, and thereby illustrate the extreme difficulties in scaling up current HIV interventions; health workers are already overburdened, and the goal of placing many more patients on antiretroviral therapy, which is labour-intensive, is impossible without a major influx of health workers. In addition to creating a "brain drain", the emigration of skilled workers can also have the effect of subsidizing education in receiving countries. Since education costs are mostly borne by the public sector, sending countries are essentially paying to educate part of the medical workforce in receiving countries. Nearly one quarter of physicians in the US are foreign-trained, and the majority of these are from low-income or lower-middle-income countries [8]; thus, "subsidies" are flowing from the poor to the rich. The amount of benefit that receiving countries obtain can be estimated from the cost to educate physicians that is avoided: in the US, it costs approximately $372,000 to educate one physician; this figure suggests that these "subsidies" total into the hundreds of millions [8].

In general, the patterns of migration are dominantly to countries with better living standards, salaries and working conditions. Wage gaps and demography are key determinants, both historically and in the present day [9]. Eastwood et al. [10] describe a "medical carousel" of migration in which doctors continuously migrate to these countries and whereby vacancies that are filled in countries with the highest living and working conditions, and salaries produce changes further along the chain. For example, a doctor migrating from an urban South African hospital to take up a post in the UK provides a doctor working in a rural area in South Africa with a post in an urban area, so that the net effect of the South African doctor migrating to the UK is a vacancy in a rural hospital in South Africa. Thus, the net effect would generally be declining numbers of doctors in the 'least desirable' areas, arguably often those that need them most.

Sub-Saharan Africa has, over the last 50 years, consistently had higher rates of out-migration than in-migration; some of the dynamics of the extremely variable migration patterns are described in Lucas [11]. Over approximately the same time period, Britain has played host to an increasing number of immigrants [12] and has been identified as having a pivotal role in the drain of health workers from HIV/AIDS-ravaged sub-Saharan Africa [10]. Large increases in net immigration of foreign citizens to Britain have been attributed, in part, to more permissive UK immigration policies, where there has been a considerable increase in the allocation of work permits and relaxation of controls on non-economic immigration since the late 1990s [12]. Some of the UK's health sectors are greatly dependent upon foreign-trained nurses, and so these are motivated to sponsor extended work permits [3]. Increasing dependence on foreign-trained health professionals is not limited to the UK and skilled worker migration is not limited to health professionals. A major group of migrants from southern African countries are natural scientists and engineers [5].

Reasons for migration are most commonly expressed in terms of push and pull factors. With respect to health professionals, Eastwood et al. [10] identified push factors (including lack of opportunities for postgraduate training, poor remuneration, poor working conditions, civil unrest and personal security) and pull factors (including opportunities for career advancement, greater financial rewards, improved working conditions, and attraction to centres of medical and educational excellence) related to living and working conditions as primary reasons for migration. Another push factor that has been cited as a reason for leaving sub-Saharan Africa was the poor quality of postgraduate training at home [13]. Crime and personal safety ranked high among push factors in South Africa, while more traditional economic factors, such as taxes, cost of living and public services were cited in Zimbabwe [14]. Political corruption, poor infrastructure and low standards of living were cited as push reasons in Ghana and Nigeria [15]. There are other reasons to migrate, such as the desire to join family abroad, and many immigration policies provide specific routes for this type of immigration. Evidence suggests that push factors are much more important in driving migration than pull factors [14].

Migration policies in receiving countries have been found to play a role in stemming migration; evidence suggests that migration policies are constraints to the level of migration [16,9]. However, even with very restrictive migration policies, migrants are often able to legally enter these countries [16], even during times when policies are tight. For example, in 2001, the UK implemented a code of practice that limited government recruitment of health professionals from developing countries. This policy, however, did not apply to the private sector, and so migrants entered the UK via the private sector and subsequently found employment in the National Health Service, making this policy basically ineffective [3]. Immigration policies, such as the one in the UK, are sometimes suggested as part of a solution to stem the outflow of health workers from sub-Saharan Africa: the more difficult it is for health workers to migrate, the smaller the outflow should be.

The reality is, however, that as long as shortages exist across the developed and developing world, there will always be net migration towards areas where wages and salaries are higher, the standard of living is better, and where there are better professional opportunities. If some receiving countries restrict access, migrants will choose other countries or will find other routes to migrate to the countries that they wish to settle in. Since migration is seen as a basic human right, there are ethical questions about discouraging migration from countries that are politically unstable, have low living standards, or offer little in terms of professional advancement or recognition, and especially to discourage it more for a certain professional group. A dire consequence of such policies might be that fewer people choose to become health workers: there is increasing evidence that people choose health careers for the possibility to migrate, not because they value the profession [17]. The most obvious example of this is that of the Philippines, where nurses are educated for migration, and where a domestic shortage of nurses exists as most Filipino nurses migrate to other countries. From a global perspective, the world could not afford having even fewer individuals enrol in health careers, since current educational placements are hardly sufficient to cover global demand.

There are other problems with health care systems that are not caused by migration. The OECD [18] reports that there are many registered nurses in South Africa who are inactive or unemployed, and that there are problems allocating health workers between public and private sectors, bringing to the surface a myriad of other problems that need to be solved. However, even if all the nurses in South Africa were employed, the total would still fall far short of what is needed.

The shortage of health workers that is causing difficulties achieving the target to halt the spread of HIV/AIDS is part of a fairly global problem of underinvestment in health professionals. The perspective taken to solve the problem must also be a global one. As long as vacancies exist in countries with better living and working conditions, and wages, migration will be towards these; this is true for other professions as well.

Discrepancies are not restricted to developed versus developing countries. Within developed countries, there are substantial differences in nurse-to-patient and physician-to-patient ratios, most notably between rural and urban areas. For example, in 2004, 9.4% of physicians in Canada were located in rural areas, while 21.4% of the population resided in these locations; it is estimated that the number of additional family physicians needed in rural areas to equalize physician-to-population ratios between rural and urban areas is 1,308, or over 25% of the current rural physician workforce [19]. There are a variety of reasons why health professionals prefer urban areas to rural areas. The largest sources of dissatisfaction for rural doctors in Saskatchewan, Canada, were that they were not able to take enough time off work and that they were required to work long hours with burdensome on-call requirements [20]. Almost 25% of rural doctors in Saskatchewan were on call 24 hours a day, 7 days a week, though call intensity and number of patients seen was small, suggesting that long hours are not rewarded with adequate compensation [20]. Similar reasons for dissatisfaction have been documented for other provinces in Canada [21], and for other countries [22,23]. Other sources of dissatisfaction for rural doctors include insufficient access to urban amenities [24], lack of professional contact with colleagues, downsizing of hospital facilities and not enough opportunities to continue medical education [25]. Higher job dissatisfaction has been associated with lower retention rates [24,25]. Consequently, recruiting doctors to, and retaining them in, rural areas has become a major policy focus.

This has provided international medical graduates with relatively easy access to jobs in countries that actively recruit to fill rural vacancies. In 2004, 26.3% of physicians in rural Canada were international medical graduates, compared with 21.9% in urban areas, though there were large variations across provinces and territories (10.9% in Quebec; 52.1% in Saskatchewan) [20]. In Saskatchewan, 40.4% of all rural physicians obtained their first medical degree in Africa [20]. In 1997, Alberta successfully recruited 40 South African doctors to rural communities [26]. However, the problem is not limited to rural-urban discrepancies: many inner-city hospitals in the US were found to rely heavily on foreign medical graduates to treat the poor [27].

Regional disparities also exist in sub-Saharan Africa, and for similar reasons. Doctors' job dissatisfaction in hospitals in rural Africa were linked to substantial after-hours work, large workloads and a perceived lack of management support [28]. Interventions that have been identified as a way to retain doctors in rural areas in South Africa include better remuneration, opportunities for career progression and continuing medical education, improving working conditions, increasing leave allocations, better hospital environments and recreational facilities, and assistance to the doctors' families [29].

Although migration from a developing country to a developed country is substantially more costly (both financially and emotionally), many reasons for it are similar to those for migration within countries. As a result, a "global hierarchy" of health-sector jobs exists, in which jobs in large urban centres in developed countries are at or near the top, while jobs in rural areas of the poorest countries at the bottom. General migration patterns are towards the top of this hierarchy, and it seems that moving up even in smaller steps is desirable, as evidenced by the large numbers of international medical graduates in rural and inner-city poor areas in developed countries. In developed countries where immigration is facilitated for migrants who promise to settle in an area of high need (e.g. a rural area), there is little evidence that such policies alone create long-term sustainable work forces in these areas, as migrants eventually move on to better jobs. And so migration continues upward until all jobs are filled. Consequently, the shortage of health workers in rural sub-Saharan Africa is closely intertwined with vacancies in developed countries, and as long as shortages exist there, they will be a significant pull factor for health professionals in developing countries. At the same time, as long as working conditions remain poor at the bottom of the hierarchy, they will continue to be a strong push factor.

Hence, the only ethical and feasible long-term solutions are improving working conditions that would encourage potential migrants to stay and the diaspora to return, and increasing educational places in countries with shortages. A huge investment in educational places worldwide will have to be made, as it is obvious that, given current numbers, most countries will not graduate enough health professionals to fill their own vacancies. In Africa, the number of health professionals currently required to deal with the AIDS epidemic alone is far greater than the numbers that are graduating.

In addition, solutions must be implemented that will work towards improving working conditions, and many of these can be relatively inexpensive and easy to put into practice. A report [30] that describes seven successful initiatives - which led to higher retention of health workers, improved access to health care in rural areas and to more efficient uses of limited resources - highlights, among other things, the importance of small improvements in working conditions. The initiatives included providing transportation from rural areas to cities, and providing accommodation in cities, where health workers can see or have access to family and friends; offering better wages; making scholarships available to local students to allow them to become health workers; educating members of local communities to help and support health workers and patients; providing training to non-professional staff to perform or help with basic procedures; and providing separate health facilities to health workers that are HIV-positive so that they avoid being stigmatized in their place of work.

In 2006, the World Health Organization launched an initiative to strengthen human resources for health, involving strategies under three headings [31]: "treat" (including protection from HIV transmission in the health care environment); "train" (including shifting tasks to less-specialised health workers, e.g. from nurses to community-health workers, and incorporating AIDS-specific training, which might also help retain health workers); and "retain" (including improving the quality of the work environment). These types of initiatives, when appropriate to their setting, are crucial to the broader goal of creating a sustainable health workforce; in addition, they address problems in poor countries that are often very similar to the problems that developed nations are facing in reducing regional disparities. In short, a major part of the solution to recruiting and retaining health professionals in underserviced areas lies in providing them with better working conditions, and to work towards better equalization in working conditions among all regions of the world, as much as that is possible.

Increasing the health workforce and improving working conditions are supply-side issues. Demand-side issues should also be addressed, because these can reduce some of the pressure on health systems. For example, while making antiretroviral therapy available to all AIDS patients should remain a priority, other measures that are less labour intensive and that have some short-term benefits could be scaled up. One such possibility is mass treatment for sexually transmitted diseases, leading to the short-term benefits of having lower prevalence of illnesses and a fairly immediate reduction in HIV prevalence, and (hopefully) a boost in the morale of healthcare workers. Oster [32] has shown that differences between the transmission rate of the HIV virus in South Africa and the rate in the United States can be almost entirely explained by the much higher prevalence of sexually transmitted infections in South Africa. Korenromp et al. [33,34] have shown in simulations and community-based trials that treating individuals for sexually transmitted diseases can result in substantial decreases in HIV infection rates in certain communities. Other programs might include more routine use of nevirapine, which has been found to nearly halve all perinatal HIV infections, and was cost-effective in resource-limited settings [35]. Some of these programs can be implemented in certain settings without substantial investments in infrastructure and labour, and will help lower morbidity and mortality. And so, as extensive investments in increasing the size of the global health workforce and improving allocation of labour by improving working conditions are made, healthcare systems could become considerably more manageable in the future.

The issue is one of global supply and demand: supply shortages in developed countries that contribute to the demand for foreign-trained health professionals must be addressed by these countries through increased investments in education and improving allocation of their own workforce; supply shortages in developing countries must also be met by increasing investments in education, while efforts must be taken to retain their health workers; demand for health workers, in general, and in countries ravaged by HIV/AIDS in particular, should be reduced by instituting as many health-improving interventions as possible; these must take into account the current ability of the country or region to administer them. Implementing policies that restrict migration of health workers is not the answer, because they do not address any of the underlying problems that cause migration. Such policies would go against the incentives of migrants and so would likely be ineffective in achieving the goal to increase the health workforce in areas with severe shortages. And they are not aligned with protecting basic human rights.

## Competing interests

The author declares that they have no competing interests.
